# Supplementary material for: Inhibition of Respiratory Syncytial Virus Infection by Small Non-Coding RNA Fragments
Source: Int J Mol Sci. 2022 May 26;23(11):5990. doi: 10.3390/ijms23115990 (PMC9180592; doi:10.3390/ijms23115990)
Supplement: Supplementary file 1 [file ijms-23-05990-s001.zip › ijms-1720530-supplementary.pdf]

Inhibition of Respiratory Syncytial Virus Infection by small non-coding RNA fragments

Sandra Axberg Pålsson[1*], Vaishnovi Sekar[2], Claudia Kutter[3], Marc R. Friedländer[2], Anna-Lena Spetz[1*]

SUPPLEMENTARY FIGURES

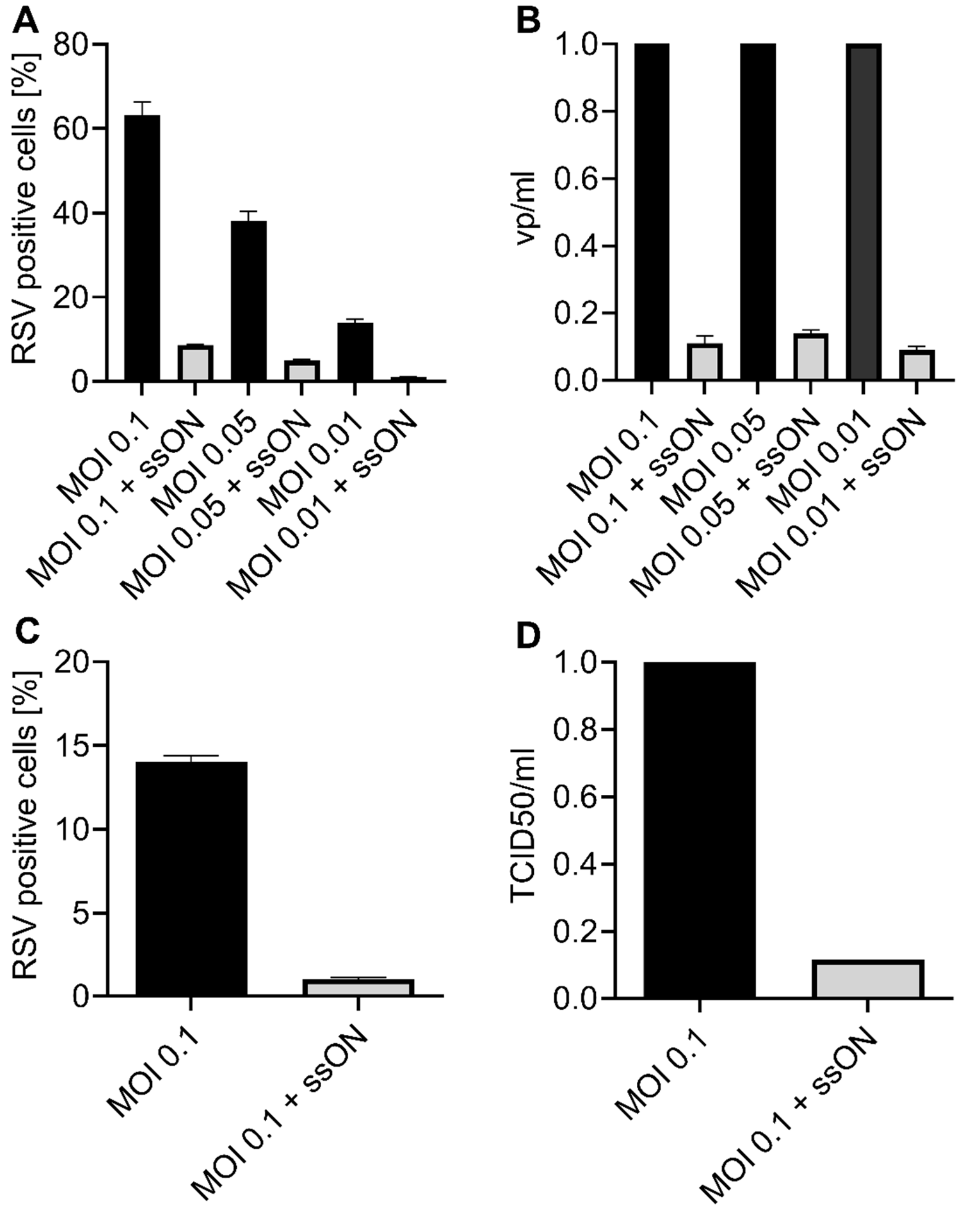

Supplementary Figure S1. Method validation. Cells were treated with 35 mer ssON (1 μM) 2 h prior to RSV infection at indicted MOIs. (**a**) The frequencies of infected cells were measured 72 h post infection using flow cytometry. (**b**) The flow cytometry data were verified using RT-qPCR to measure viral content in the supernatant. (**c**) The infectivity of the virus was determined by performing a $TCID_{50}$ assay wherein the supernatants (24 h after

infection) from cell cultures in (**c**), were used in a (**d**) TCID50 assay which was assessed 5 days after transfer. The results in B and D were normalized to the RSV control. Data is from one experiment conducted in duplicate.

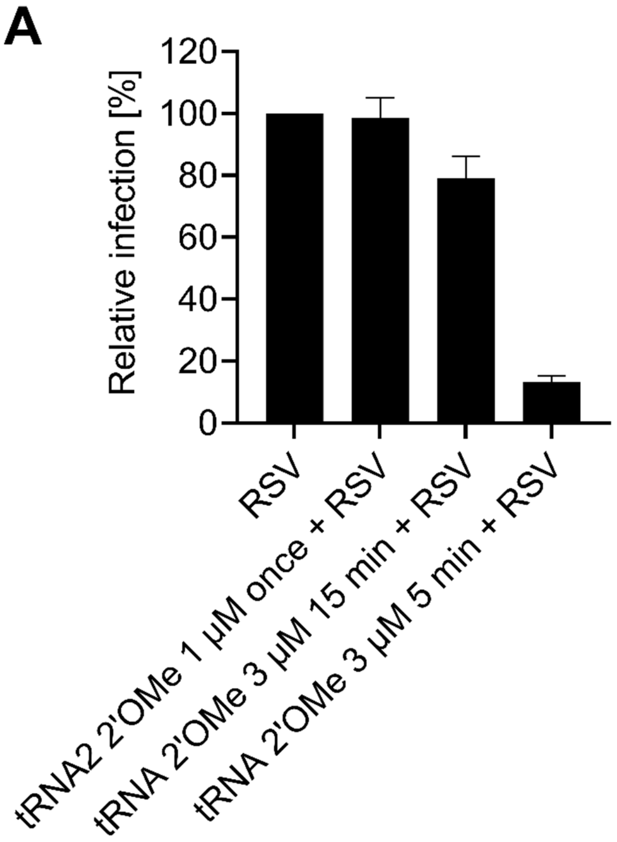

Supplementary Figure S2. sncRNAs with naturally occurring modifications can inhibit RSV infection in A549 cells. Cells were treated with extracellular addition of tRNA2 2′OMe (1 µM) 30 min prior to RSV infection using MOI 1 ("tRNA2 2′OMe 1µM once"). Alternatively, cells were treated with tRNA 2′OMe (3 µM) for 15 min prior to infection with new addition of tRNA 2′OMe (3 µM) every 15 min during the 45 min incubation with RSV ("tRNA2 2′OMe 3µM 15 min"). Lastly, cells were treated with tRNA 2′OMe (3 µM) for 5 min before infection and new tRNA 2′OMe (3 µM) were added every 5 min throughout the 45 min incubation with RSV ("tRNA2 2′OMe 3µM 5 min"). After infection, cells were washed prior to resuspension in media and incubation for 24 h. The cells were subsequently stained with LIVE/DEAD® Fixable near-IR Dead Cell Stain Kit and the proportion live RSV-infected cells was measured 24 h post infection using flow cytometry. The data is presented as mean ± SEM from one independent experiment conducted in duplicate for the "tRNA2 2′OMe 1µM once" and "tRNA2 2′OMe 3µM 5 min" samples and two independent experiments conducted in duplicate for the "tRNA2 2′OMe 3µM" 15 min sample.

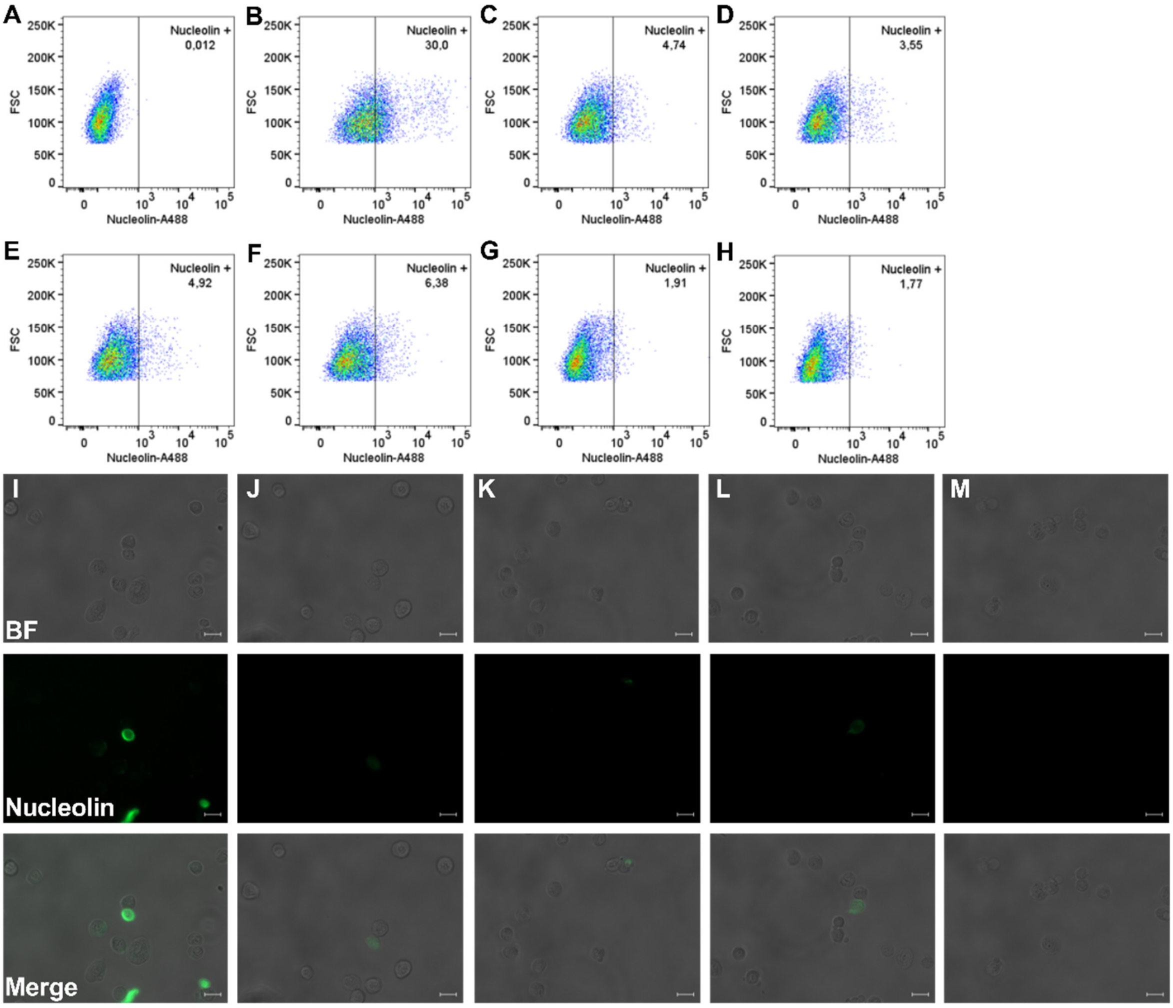

Supplementary Figure S3. Small non-coding RNAs inhibit antibody binding to the RSV entry co-receptor nucleolin.A549 cells were treated with 35 mer ssON or RNA oligonucleotides (1 µM) for 15 min followed by incubation with an anti-nucleolin antibody (1 µg/ml) for 30 min at 4°C and then stained with an Alexa 488-conjugated donkey anti-rabbit IgG secondary antibody for 30 min at 4°C. Representative flow cytometry plots of nucleolin antibody stainings are shown in (**a**) unstained negative control cells, (**b**) baseline nucleolin control cells (**c**) ssON-treated cells, (**d**) tRNA2-treated cells, (e) YRNA2-treated cells, (**f**) rRNA2-treated cells, (**g**) isotype control and (**h**) secondary antibody control. Data are representative from three independent experiments conducted in duplicate. Representative microscopy pictures showing nucleolin antibody staining in (**i**) baseline nucleolin control cells, (**j**) ssON treated cells, (**k**) tRNA2 treated cells, (**l**) YRNA2 treated cells and (**m**) rRNA2 treated cells. BF=bright field, Nucleolin=nucleolin-A488 staining, Merge=combined brightfield and nucleolin A488 staining. Data representative from one experiment performed in duplicates. Scale bar 20µM.

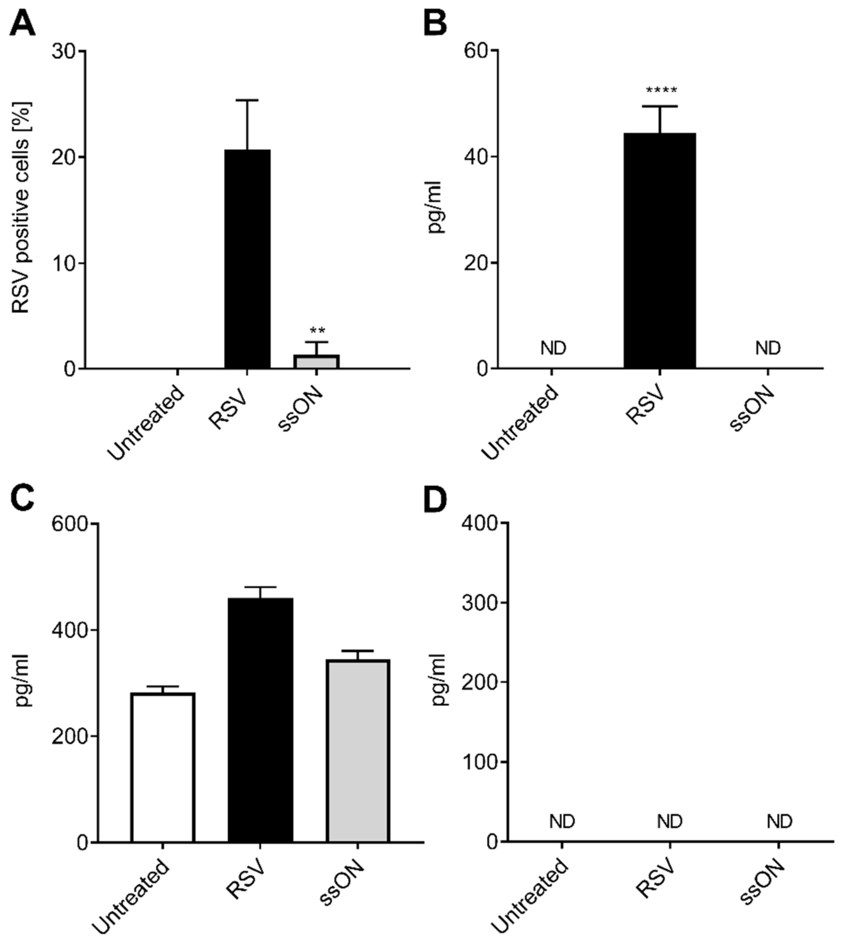

Supplementary Figure S4. Cytokine secretions in A549 cells after RSV infection and ssON treatment.A549 cells were treated with 35 mer ssON (1 μM) 2 h prior to RSV infection. (**a**) The frequencies of infected cells were measured 24 h post infection using flow cytometry. The secretions of (**b**) IL-29, (**c**) IL-8 and (**d**) INF-α in the supernatants (24 h) were measured using ELISA. ND=non-detectable. Results are presented as mean ± SEM from three experiment conducted in duplicate with two replicates per sample for (**a**) and (**b**) and one experiment conducted in duplicate with two replicates per sample for (**c**) and (**d**). Significant differences were assessed using the non-parametric Mann-Whitney test by comparing the ssON treatment to the RSV infected control in (**a**) and comparing all treatments to the untreated control in (**b**). *P*-value: ** $P \leq 0.01$; **** $P \leq 0.0001$. Lack of significance is not displayed in the figure.

Supplementary Table S1. Sequences, descriptions and IC_{50} values of RNA and DNA oligonucleotides used.

Name	Nomenclature name	Sequences	Length	Approximate IC_{50} (nM)	Chromosome (hg38)	Start position	End position	Description	ID
ssON		GAAGTTTTGAGGTTTTGA AGTTGTTGGTGGTGGTG	35	92*					
YRNA 1	C8RNA:chr7:1 48941487-148941520	AGUUGGUCCGAGUGUU GUGGGUUAUUGUUAAA AA	34	53	chr7	14894148 7	14894152 0	Human 4.5S (C8) RNA. Y RNA {clone Y5-125, small RNA known as Ro RNA} [human, HeLa cells, Other Mutant, 118 nt].	K01106.1, S76546.2
YRNA 2	Y4RoRNA:chr7 :148963314-148963347	GGCUGGUCCGAUGGUAG UGGGUUAUCAGAACUU	33	85	chr7	14896331 4	14896334 7	Human hy4 Ro RNA (associated with erythrocyte Ro RNP's).	X57566
rRNA1	28SrRNA:chr21 :8213888-8213924	CGCGACCUCAGAUCAGA CGUGGCGACCCGCUGAA UUU	37	169	chr21	8213888	8213924	28S rRNA mapping to chr21.	NG_055263
rRNA2	5.8SrRNA: UnassembledC ontig:112024-112053	CGACUCUUAGCGGUGGA UCACUCGGCUCGU	30	234	Unassembled genome contig	112024	112053	Human 5.8S ribosomal RNA.	J01866.1
tRNA1	tRNA-Gly:chr16:7078 8694-70788727	GCAUUGGUGGUUCAGU GGUAGAAUUCUCGCCUG C	34	72	chr16	70788694	70788727	TPA: Homo sapiens tRNA-Gly-GCC-5-1 gene. TPA: Homo sapiens tRNA-Gly-GCC-3-1 gene. TPA: Homo sapiens tRNA-Gly-GCC-2-1 gene.	HG983819, HG983817, HG983811
tRNA2	tRNA-Glu:chr6:12578 0285-125780318	UCCCUGGUGGUCUAGUG GUUAGGAUUCGGCGCU	33	54	chr6	12578028 5	12578031 8	Human Glu-tRNA. Homo sapiens RNA, tRNaseZL-interacting RNA A2.	M31637 AB330770
15 mer RNA		GGUUUUGAAGUUGUU	15						

*The IC_{50} value for ssON was presented in a previous study[12].
